# Proteolytic Processing of CD44 and Its Implications in Cancer

**DOI:** 10.1155/2021/6667735

**Published:** 2021-01-06

**Authors:** Priscila Anhel Medrano-González, Osmar Rivera-Ramírez, Luis Felipe Montaño, Erika P. Rendón-Huerta

**Affiliations:** ^1^Lab. Inmunobiología, Depto. Biología Celular y Tisular, Facultad de Medicina, UNAM, Mexico, Mexico; ^2^Posgrado en Ciencias Biológicas, Unidad de Posgrado, Edif. D, 1 piso, Circuito de Posgrados, Ciudad Universitaria, Coyoacán, 04510 Mexico, Mexico

## Abstract

CD44 is a transmembrane glycoprotein expressed in several healthy and tumor tissues. Modifications in its structure contribute differently to the activity of this molecule. One modification that has provoked interest is the consecutive cleavage of the CD44 extracellular ectodomain by enzymes that belong mainly to the family of metalloproteases. This process releases biologically active substrates, via alternative splice forms of CD44, that generate CD44v3 or v6 isoforms which participate in the transcriptional regulation of genes and proteins associated to signaling pathways involved in the development of cancer. These include the protooncogene tyrosine-protein kinase Src (c-Src)/signal transducer and activator of transcription 3 (STAT3), the epithelial growth factor receptor, the estrogen receptor, Wnt/*β*catenin, or Hippo signaling pathways all of which are associated to cell proliferation, differentiation, or cancer progression. Whereas CD44 still remains as a very useful prognostic cell marker in different pathologies, the main topic is that the generation of CD44 intracellular fragments assists the regulation of transcriptional proteins involved in the cell cycle, cell metabolism, and most importantly, the regulation of some stem cell-associated markers.

## 1. Main Text

### 1.1. Structure and Function of CD44

**Figure 1:**
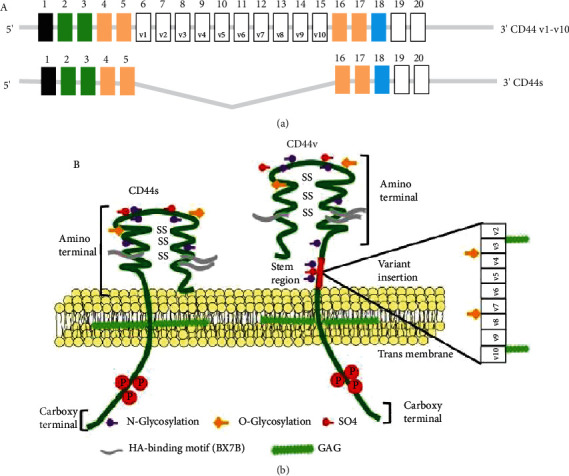
Structure of CD44. (a) The CD44 gene encodes 20 exons, of which exons 6 to 15 correspond to variable exons (v1-v10). The standard isoform only contains the constant exons. (b) CD44 is composed of an extracellular domain, a stem region, a transmembrane domain, and a cytoplasmic domain. CD44 is subject to posttranslational modifications like N- or O-glycosylation and sulfation. The extracellular domain contains conserved disulfide bridges and two BX7B domains, which are essential for hyaluronic acid (HA) binding. The cytoplasmic domain contains phosphorylation sites that regulate the interaction between CD44 and the cytoskeleton through linker proteins (modified from Misra, S. et al. *Frontiers in Immunology* 6:201, 2015) [20].

CD44 is a cell surface adhesion molecule involved in cell-cell interactions, cell adhesion, and migration [1]. The main ligand of CD44 is hyaluronic acid (HA), a polysaccharide abundantly present in the extracellular matrix of mammals, yet it can bind to other components of the extracellular matrix and perform different functions depending on the structure of the protein it binds to [2, 3]. It is composed of a distal extracellular amino-terminal domain (ECD), a stem region, a transmembrane domain (TM), and an intracellular cytoplasmic carboxy-terminal domain (ICD) (Figure 1).

The CD44 gene encodes 20 exons, of which exons 6 to 15 correspond to variable exons (v1-v10). There is one standard isoform, designated CD44s and splice variants that contain variable exons, designated CD44v. The ECD structurally corresponds to a globular protein stabilized by disulfide bridges between three pairs of cysteine residues [2]. Carbohydrate chains, usually glycosaminoglycans (GAG) bind to this domain, which confer CD44 with a negative electric charge and rigidity [4]. The stem region is where the variable exons are inserted in the CD44v isoforms. The TM domain has an important role in the localization of CD44 on the cell surface [5], as a conserved cysteine residue in this region (cysteine 286) promotes its homodimerization and binding to HA [6, 7]. The ICD interacts with actin filaments through ezrin-radixin-moesin (ERM) proteins, which bind to CD44 through their amino-terminal FERM domain and to the actin cytoskeleton through their carboxyl-terminal domain [8]. The association between CD44 and the cytoskeleton allows the modulation of cell form and cellular motility [9]. The association between the ICD of CD44 and ERM proteins induces changes in the cytoskeleton architecture and allows the transduction of some signaling pathways since CD44 also interact as a coreceptor for a great amount of receptors [10–13]. For example, the ICD participates in the activation of Ras through the recruitment of ERM proteins in the c-Met signaling pathway [10]. Nevertheless, associations of proteins such as a CD44-associated phosphatase 2A with the ICD, where ERM proteins do not have a clear role, have been reported in leukemic T cell apoptosis [14].

The crystallographic structure of the CD44 hyaluronic acid binding domain (CD44 HABD) has shown that it is composed of two alpha helices and two beta sheets constituted by six and three strands, respectively. After the binding of CD44 HABD to HA, a rearrangement in the beta strands, a *β*9 present in the HA-unbound state of CD44 HABD, is not present anymore, and a disorder of the molecule structure occurs [15].

CD44 alternative splicing generates a great amount of isoforms [16]. Ten of the exons contained in the gen are expressed in all the isoforms (constant exons), while the remaining ten central exons (variable exons) are added or eliminated in the stem region in different combinations in different isoforms (Figure 1). The standard isoform (CD44s) lacks all the variable exons and is expressed in most of the cells of vertebrate animals, while the variant isoforms (CD44v) are expressed only in some cells under specific conditions [17]. The ICD can be subject to alternative splicing too, since the differential use of exons 19 and 20 generates a short version with 3 amino-acid residues, and a longer version with 70 amino-acid residues, being the longer version the more abundant [1]. Additionally, different posttranslational modifications generate additional diversity in its structure. Interestingly, different isoforms of CD44 are known to acquire different functions depending on the variable exons included in its structure since they induce conformational changes that allow the new isoforms to generate new binding sites; for example, the sequence that is encoded by the variable exon 6 has a binding site for the hepatocyte growth factor (HGF) and for the vascular endothelial growth factor (VEGF) [18] while the variable exon 3 has an heparan sulfate-binding site that allows it to bind to some other growth factors such as the fibroblast growth factor (FGF) or the epidermal growth factor (EGF) [19].

In mammals, CD44 is expressed in hematopoietic lineage, endothelial cells, and epithelial cells [21]. The ECD has a highly conserved region (85% identity), which corresponds to exons 1-5, and a variable region where variable exons are inserted. The membrane-proximal region, encoded by exons 16 and 17, is less conserved (35%), while exon 18, which encodes the TM region, is 100% conserved [2]. The genomic structure of CD44 is highly conserved between humans and mice, and the length of the introns in the regions involved in alternative splicing is almost identical [22].

In humans, CD44 is expressed in numerous tissues, including the central nervous system, lungs, epidermis, liver, and pancreas, among others. The variable isoforms of CD44 have a more restricted distribution than CD44s, which suggests that the alternative splicing of this protein is a highly regulated process [23]. Additionally, the expression of distinctive isoforms of CD44 during different embryonic stages has been reported; for example, CD44v9 isoform is predominantly expressed in the epidermis, trachea, lungs, thyroid gland, mesonephric ducts, and paramesonephric ducts, while CD44v6 isoform can be detected in the epidermis and tracheas of 10th week human fetuses. In human fetal thymus, CD44s is expressed in the cortical region, while the CD44v9 isoform is expressed in medullary cells [24], thus establishing that the expression of different isoforms of CD44 can be tissue-specific and stage-specific. In T cells, CD44 interacts with CD4, forming a complex where, presumably, CD3 and the TCR are recruited to mediate the activation of T cells [25]. CD44 can be distributed in lipid rafts, where several molecules are recruited to facilitate signal transduction. The localization of CD44 in lipid rafts is positively regulated by the palmitoylation of cysteine residues, while the presence of phosphatidylinositol 4,5-bisphosphate (PIP_2_) decreases its affinity for lipid rafts [26]. Once located in lipid rafts, CD44 can suppress the binding of ERM proteins to the ICD, and in T cells, the binding of CD44 to HA can be regulated, participating in the regulation of adhesion and migration processes [27].

### 1.2. CD44 Expression and Cancer Stem Cells

In the late 1990s, a small subpopulation of cells in the hematopoietic and tissue cancers was identified [28]. This cell subpopulation shares characteristics with progenitor cells and stem cells, mainly its self-renewal property among other characteristic, so they have been called *cancer stem cells* (CSC). There is a strong belief that this subpopulation is responsible for the initiation, progression, metastasis, and tumor recurrence [29]. Among the different molecular markers that have been used to identify CSCs, CD44 stands out (Table 1) [30]. The expression of CD44 in cancer cells and in CSC of several types of tumor substantiates the highly important role of CD44 in the development and progression of cancer.

### 1.3. Interaction of CD44 with Surface Receptors

Hyaluronic acid, an anionic nonsulfated glycosaminoglycan, is the primary CD44 binding molecule (Wang L, Methods Mol Biol 2018). CD44 also interacts with other highly relevant receptors such as TM4SF5, a tetraspanin involved in G1/S progression phase [31], osteopontin [32, 33], or CD74, the receptor for the macrophage migration inhibitory factor that induces the cleavage and release of its cytosolic extracellular domain which regulates cell survival [34]; this interaction activates signaling pathways involved in survival and proliferation of the cancer cells [35]. In hepatocarcinoma cells, a physical interaction between CD44 and TM4SF5 through their extracellular domains was involved in the activation of the protooncogene tyrosine-protein kinase Src (c-Src)/signal transducer, and activator of transcription 3 (STAT3) signaling [36]. It is becoming clear that some CD44 isoforms such as CD44v3 or v6 are recruited in a ligand-dependent manner as coreceptors in the epithelial growth factor or estrogen receptor signaling pathways [37]. CD44 interacts with small hyaluronic acid oligosaccharides [38]; furthermore, CD44 can bind *α*5*β*1-integrin, lymphocytes mannose receptors, and a4b1-integrin a well-recognized receptor for the vascular cell adhesion molecule VCAM-1 [39–41]. These interactions point to the importance of CD44 in the interaction and cellular communication with extracellular media components.

### 1.4. Enzymatic Cleavage of CD44

**Figure 2:**
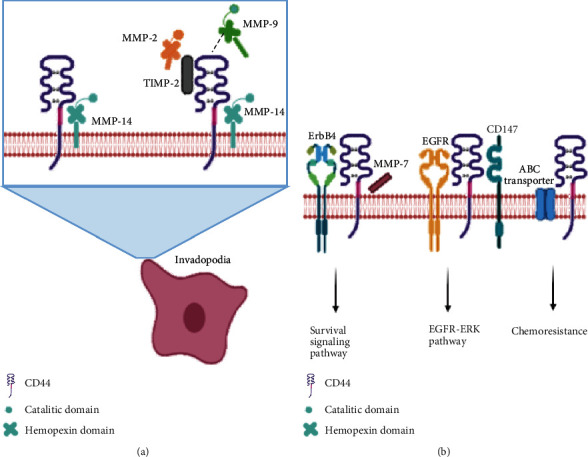
CD44 and interaction with soluble and/or cell membrane ligands. (a) The interaction between CD44 and MMPs directs the latter to the cell front of migrating cells, the so-called invadopodia, simplifying the degradation of substrates present in the extracellular matrix. (b) CD44 interacts and forms complexes with proteins expressed on the cell surface such as the enzyme Erb-B2 receptor tyrosine kinase, epidermal growth factor receptor, or ABC transporter all of which stimulate signaling pathways that promote survival, proliferation, or chemoresistance.

CD44 binds some metalloproteinases (MMPs), like MMP-2, MMP-9, MMP-14, MMP-15, MMP-16, MMP-24, and MMP-25, through their hemopexin domain in lamellipodia edges [42, 43] (Figure 2). CD44 interaction with MMP-9 on the cell surface of melanoma cells promotes the degradation of collagen IV and cell invasion [44]. Clustering of CD44 allows the retention of MMP-9, promoting its proteolytic activity on the membrane [45], but also and interestingly, the active form of MMP-2 coprecipitates with CD44, indicating its involvement in the enzyme activation [46].

CD44 forms a platform for the assembly of several MMPs with their substrates [47]. In normal mouse breast and uterine epithelium, the isoform CD44v3 recruits the active form of MMP-7 and the precursor of EGF, which bind to heparan sulfate-binding sites present in the exon v3; subsequently, ErbB4 is recruited, and survival signaling pathways are activated [48]. On the contrary, in some cancers such as in leukemia, MMP-2 and MMP-9 bind to CD44 in a complex where MMP-14, interacting with CD44v6, cleaves and activates MMP-2, inducing the degradation of the extracellular matrix [49]. The upregulation of CD147, also known as extracellular matrix metalloproteinase inducer, in epithelial cells leads to the formation of lipid raft-associated complexes composed of CD147, EGFR, and CD44, which activate the EGFR-Ras-ERK signaling pathway, facilitating chemoresistance processes, cell proliferation processes, and the activation of antiapoptotic signaling pathways [50]. However, other receptors with tyrosine kinase activity, like ErbB2 and ATP-binding cassette (ABC) transporters, may participate (Figure 2).

The interaction between these enzymes and CD44 generates the cleavage of its ECD, which is involved in the secretion and activation of MMP-9 [51]. The interaction between MMP-14 and CD44 promotes cell migration through a mechanism that is dependent on the cleavage of the ECD [52]. Interestingly, although CD44 binds to MMP-17 and MMP-25, CD44 is not cleaved [43]. In different models, ADAM10 and ADAM17 have been involved in the cleavage of CD44, mainly in melanoma cells [53, 54]. Most of the proteases that are involved in the cleavage of the ectodomain of CD44 are members of the MMPs or the ADAMs; however, the participation in this process of other enzymes such as cathepsin [55] or putative chymotrypsin-like serine proteinases [56], which are also able to cause the release of ECD, remains to be clarified.

### 1.5. Sequential Cleavage of CD44

CD44 can be found in three phases: a membrane receptor, an integral component of the extracellular matrix, and a soluble fragment present in fluids, where serine proteinases and MMPs are involved [57]. As mentioned before, cleavage of CD44 releases ECD fragments with a molecular weight in the 25 KDa range [58], suggesting the participation of several enzymes [35] as different cleavage sites can be exposed. In osteosarcoma cells, the spontaneous generation of 90 and 70 KDa ECD fragments has been observed, but when MMP-14 expression was induced, an additional 50 KDa fragment was also found. The latter strongly suggests that MMP-14 generated a fragment that was independent of posttranslational modifications [42]. The release of the ECD leaves 18-25 KDa truncated fragments in the cell membrane in articular chondrocytes of osteoarthritis patients and in prostate cancer cells [59, 60].

The cleavage of the ECD is a necessary step needed for the generation of the ICD by a mechanism called *regulated intramembrane proteolysis* (RIP) [16]. The latter is an evolutionarily conserved process characterized by the cleavage of transmembrane proteins and the release of cytosolic fragments [58, 61]. The CD44 ICD fragment is generated through the cleavage of the TM domain, by an enzyme complex formed by presenilin1-presenilin 2-*γ*-secretase [62, 63]. After the activity of this complex is exerted upon its substrate, fragments with different molecular weights are released. Nevertheless, there are some instances where a dual intramembrane cleavage mechanism generated by the secretase complex generates small CD44 peptides (a major 16 KDa fragment and 3 minor fragments of roughly 5 KDa, also called CD44 *β*) [59, 64], or as observed in breast cancer cell lines, small 17 KDa fragments can be generated from the ICD spontaneously [65]. The ICD fragments are able to translocate to the nucleus and promote the transcription of target genes through the 12-O-tetradecanoylphorbol-13-acetate-responsive element (TRE), or cooperate with CBP/p300 and enhance transcriptional activity [58] (Figure 2).

### 1.6. Mechanisms that Promote the Cleavage of CD44

The cleavage of the ectodomain is regulated by phorbol esters, suggesting an important role for protein kinase C (PKC) [66]. The treatment with phorbol esters or the use of an anti-CD44 antibody (mAb IM7) induced the cleavage of the ECD in mouse monocytes; this cleavage was accompanied by changes in the structure of actin filaments, in a process mediated by the activation of Rac1 and Cdc42 [67]. A mechanism has been described, where 12-O-tetradecanoylphorbol-13-acetate (TPA) and ionomycin (a calcium ionophore) induce an MMP-mediated cleavage through two different pathways: the first pathway is activated by the influx of Ca^+2^ and is independent of PKC activity, while the second pathway is activated by TPA and involves the participation of Rac [68]. The influx of Ca^+2^ regulates the interaction between calmodulin and ADAM10, inducing its activation; the stimulation with TPA leads to the activation of PKC and Rac, inducing the activation of ADAM17 [69].

The cleavage of CD44 ECD is also regulated by cytokines such as interleukin 1 [59], TGF*β*1 [70] or interferon *γ* [71], and bacterial- and leukocyte-derived proteinases [57, 72], all of which induce the expression of MMP-14 and the release of the ECD [73].

Posttranslational modifications also regulate the cleavage of CD44. In melanocytes, a full or partial O-glycosylation of four serine-glycine motifs located in the membrane-proximal region is required for the spontaneous cleavage of the ECD; mutations in these sites impair this cleavage [74]. Interestingly, mutations in certain regions or posttranslational modifications in the ICD might impair the homodimerization of CD44 and impair the cleavage of the ECD, since they regulate the access of proteases to their recognition sites by inducing conformational changes [75, 76].

### 1.7. Cleavage Sites of the ECD

Three cleavage sites have been identified: Gly192-Tyr (CS1), Gly233-Ser (CS2), and Ser249-Gln (CS3). Cleavage at CS1 and CS3 happens during normal physiological processes. CS1 is recognized by MMP-14 in vitro, and it has been suggested that MMP-15, MMP-16, and MMP-24 may recognize CS1 and CS2; CS3 is thought to be recognized by a member of the ADAMs family of proteases [77]. There is evidence that MMP-14 also recognizes Arg186-Ser and Thr163-Asn sites [52], probably secondary to highly variable patterns of glycosylation in CD44; this variability could interfere with the recognition of the proper sites. ADAM10 recognizes the S230 residue. Trypsin recognizes the K196 residue, but other putative sites not exposed because of the three-dimensional structure of CD44 may exist [75].

### 1.8. Cleavage Sites of the ICD

Two cleavage sites have been identified: Ala278-Leu279 and Ile287-Ala288 [64]. The amino-acid residues 288-324 in CD44 composed the released fragment of ICD [58]. The use of a mutant CD44, whose residues 287-290 were deleted, abolished the cleavage of the ICD [58], indicating that this cleavage site is essential. The ICD fragment thus generated is responsible for transcriptional activation. The function of a small fragment, CD44-*β*, also released by ICD cleavage is unknown, but it has been proposed that it allows the efficient removal of the remaining fraction that is anchored to the cell membrane [64].

### 1.9. ICD as a Transcription Factor

After CD44 is cleaved by *γ*-secretase, the ICD fragment accumulates in the nucleus and performs different roles. The ICD regulates the expression of the transcription factor RUNX2, and it can interact with it as a nuclear cofactor [60]. Moreover, the ICD binds to the consensus sequence CCTGCG of CD44, called *CD44-ICD response element* (CIRE), in a site that is near to the binding site of RUNX2 in the promoter of MMP-9, where it regulates its expression; additionally, the CIRE sequence is present in some Hif1*α*-regulated genes thus regulating its expression under normoxic conditions, independently of Hif1*α* [78]. Under hypoxic conditions, the ICD can bind to HIF-2*α*, but not to HIF-1*α*, and induce its stabilization, which enhances the activation of HIF target genes [79].

The ICD is able to regulate the transcription of genes that have TPA response elements, including CD44, leading to a positive feedback [58]. Some ICD early responsive genes encode enzymes of the glycolysis pathway, such as ALDOC, PDK1, and PFKFB4 [78]. The ICD regulates the expression of PFKFB4 by interacting with the promoter of CREB [80]. Additionally, the ICD activates the transcription of interferon-induced genes such as gamma-interferon-inducible protein 16 (IFI16), interferon-induced transmembrane protein 3 (IFITM3), and INF*β*, where the KR sequence of the ICD seems to be a nuclear translocation motif. Besides, the ICD binds to CREB, regulating the expression of genes, such as cyclin D1 [81]. The correlation in mRNA and protein levels between CD44 and PD-L1 demonstrated that the binding of the ICD to the regulatory sequence of PD-L1 promotes its expression [82]. Since no transactivation domains have been found yet, it is believed that the ICD requires the participation of neighboring transcription factors [83].

Interestingly, the ICD regulates and activates the expression of some transcription factors related to the maintenance of stem cell characteristics, such as SOX2 and Oct-4 [65]. The ICD is able to promote the expression of factors that are related to stemness via PFKFB4-mediated glucose metabolism under normoxic conditions [80].

### 1.10. ECD, ICD, and Inflammatory Processes

Cleavage of CD44 is involved in various pathologies. The soluble fragment of CD44 (sCD44), detected in serum of healthy humans, increases its concentration in inflammatory diseases such as systemic sclerosis, chronic periodontitis, aggressive periodontitis, endometriosis, pulmonary tuberculosis, sarcoidosis, open-angle glaucoma, and pouchitis [84–91].

Additionally, there are some instances where the proteolytic cleavage of CD44 may contribute to the development of some nonmalignant diseases. The cleavage of CD44 by a membrane type-1 MMP, in type-1 diabetes, regulates the intraislet homing of diabetogenic cytotoxic T cells [92]. The concentration of sCD44, increased in synovial fluid of patients with rheumatoid arthritis, is related to partial suppression of T cell activation [93]; a similar T lymphocyte activation that correlates with low levels of sCD44 has been established in chronic pancreatitis [94].

### 1.11. Participation of CD44, Its Variants, and ICD in Cancer

CD44 can promote, inhibit, or have no effect on cell invasion, depending on its expression levels and the activity of enzymes that regulate its cleavage [95]. Actually, it regulates migration and invasion processes depending on variant isoforms created by alternative splicing [9]. Interestingly, this alternative splicing is regulated by mitogenic or oncogenic signals [96]. Despite originally being considered a receptor for hyaluronic acid, it has been observed that the interaction of CD44 with different-sized HA oligosaccharides, which could represent a sign of cellular distress especially in malignancies [97], triggers intracellular signaling pathways [98] that lead to an increase in the expression of MMP-14, cleavage of CD44, and cell migration [99]. The cleavage of CD44 facilitates the detachment from HA in the extracellular matrix, and the ICD fragment liberated by the cleavage process is capable to induce the expression of new CD44 molecules on the cell membrane, thus facilitating the binding to other sites [100]. Similarly, CD44 makes nonmetastatic cells more metastatic [101] probably related to the isoforms that the cell expresses as several CD44v isoforms are cancer stem cell markers [102] in pancreas, laryngeal, head, neck, stomach, colon, lung, breast, ovarian, prostate, glioma, leukemia, or lymphoma cancer [17].

A comparison of healthy versus malignant tissue showed CD44 overexpression in liver, colon, esophageal, kidney, thyroid, and rectal cancer, as well as in cholangiocarcinoma and pituitary adenoma [103–108]. Nevertheless, this data should be complemented with the methylation state of the promoter of CD44 since there are instances where it is frequently hypermethylated [109, 110] and functions as a silencer of tumor progression [111]. Finally, we must not put aside the microenvironment where the tumor is developing, as there is evidence that under certain environment conditions, sCD44 competitively inhibits the binding of CD44 molecules to HA [112]. A study where ECD cleavage was induced in lung epithelium tumor cells pretreated either with oncostatin M alone or combined with TGF*β*1 generated fragments with a higher affinity for HA than cells pretreated only with TGF*β*1 [70].

Changes in the tissue expression patterns of different isoforms of CD44 can impair epithelial-mesenchymal interactions and contribute to the characteristic functional and structural disorganization found in cancer [2]. Migration of tumor cells and leukocytes involves cell adhesion mechanisms similar to those that happen during embryonic development and differentiation or those established during lymphocyte recirculation, organogenesis, and embryogenesis [24].

**Figure 3:**
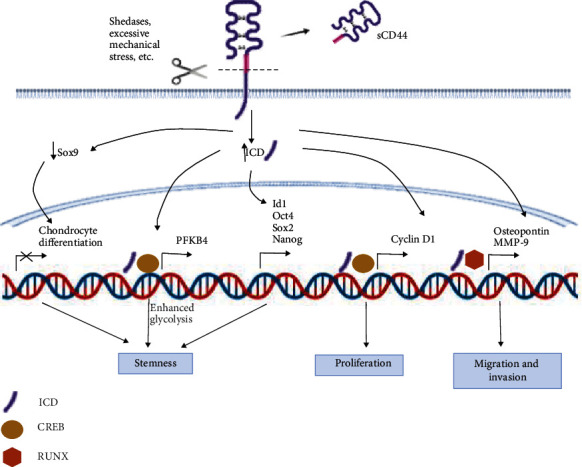
Genes activated or inhibited by the CD44 intracellular domain as reported in several epithelial cancer cells (prostate, thyroids, chondrocytes, and glioblastoma).

Downstream signal transduction is involved in cancer progression and signal transduction. Because CD44 lacks kinase activity, it can transduce signals via coupling its intracellular domain to adaptor proteins or kinases such as PI3K, NFkB, or CREB [113] and target downstream genes Survivin, Cortactin, and TGF-b2 all of which are related to cancer cell invasion [18, 114–116]. TGF-*β*2 is known to regulate epithelial-mesenchymal transition in the breast [117]. In oral cancer, CD44v4 is associated to chemoresistance to cisplatin via the activation of the MEK/ERK1/2 pathway whereas CD44v6 is associated to invasiveness via the inactivation of the PI3K/AKT/GSK3B pathway [118] as it regulates MMP-9 expression [119]. CD44v6 positive gastric cells also survive longer and have lower apoptosis after cisplatin treatment [120]. CD44 promotes tumor resistance to ROS- and chemotherapy-induced stress by regulating some of the transcription coactivators of the tumor suppressor Hippo signaling pathway that consists of a cascade of conserved kinases and transcription coactivators [121, 122]. In chronic leukemia, CD44 promotes cell survival by regulating the expression of the antiapoptotic protein MCL1 via ERK and AKT activation [123]. CD44 also targets the canonical Wnt/*β*-catenin pathway and the EMT process [124, 125]. The increase in ICD diminishes Sox9 expression in articular chondrocytes thus diminishing the expression of genes associated to differentiation and favoring the expression of genes associated to stemness [126]. ICD also binds to CREB, and the dimer binds to PFKB4, a promoter that activates glycolysis and stemness in breast cancer cells [80], whereas in thyroid cancer cells, it facilitates the recruitment of cyclin D1 and thus cell proliferation [81]. Finally, in prostate cancer, ICD forms a complex with RUNX inducing the transcription of genes associated to migration and invasion such as MMP-9 and osteopontin [60](Figure 3).

## 2. Conclusions

The cleavage of CD44 happens in normal tissues as part of physiological processes. In tumor tissues, there are increased expressions of enzymes that mediate this cleavage as well as increased levels of sCD44. CD44 is deeply involved in metastasis processes as (1) it facilitates cell adhesion to blood vessels and transendothelial migration, (2) it contributes to the maintenance of stem characteristics in tumor cells through the stimulation of key signaling pathways, (3) it confers resistance to drugs through an increased expression of the MDR1 multidrug resistance gene, and (4) it confers resistance to apoptosis by modifying the expression levels of caspase 3 and 9. Tumors overexpressing CD44v isoforms such as CD44v6, CD44v9, and CD44v10 have a poor prognosis.

It is still uncertain whether the cleavage of the ECD or modifications in the ICD that induce the cleavage of the ECD can lead to different responses. This process could be a meticulously regulated mechanism that starts with the cleavage of the ECD (induced by the characteristics of the ICD) and ends in a specific transcriptional response. However, the spontaneous generation of the ECD via nonrecognized enzymes remains to be specified. Consequently, the possible distinctive mechanisms that regulate differential CD44 cleavage and participate in the transcriptional specificity of ECD and ICD fragments remain elusive.

The precise homeostatic mechanisms that are disrupted and lead to an exacerbated cleavage of CD44 (post transcriptional modifications, changes derived by an abnormal splicing, isoforms switching, and association to other unknown molecules) in pathological conditions remain to be comprehended. Understanding what triggers and regulates the process is imperative as this is a major mechanism used by cancer cells to proliferate, migrate, and transmute into an undifferentiated phenotype. Its expression is regulated epigenetically, or by miRNAs. CD44 modulates the activity of multiple cellular signaling components [127] and plays a key role in the regulation of epithelial to mesenchymal transition. Targeting well-recognized CD44 abnormal mechanism to block aberrantly activated signaling pathways in tumor cells by antibodies, peptides, aptamers, hyaluronic acid oligomers, or chemotherapy is a current priority, but to understand the precise regulatory role of CD44 intracellular domains in this complex activation of abnormal signaling pathways is beginning to be considered a priority.

## Figures and Tables

**Table 1:** Tumors and their cancer stem cell mark.

Tumor	Cancer stem cell phenotype∗
Brain	CD44^+^/CD133^+^/NPM1^+^/CD90^+^/CD49f^+^
Breast	CD44^+^/CD24^-/low^/CD29^+^/ALDH1A3^+h^/CD164^+^
Colon	CD44^+^/CD133^+^/CD26^+^/ALDH1^+^/EpCAM^+h^
Head and neck	CD44^+^/CD271^+^
Leukemia	CD44^+^/CD123^+^/CD34^+^/CD38^−^
Liver	CD44^+^/CD133^+^/CD24^+^/EpCAM^+^
Lung	CD44^+^/CD133^+^/ALDH1^+^/CDw338^h^∗∗
Ovarian	CD44^+^/CD24^+^/CD117^+^/CD133^+^/ALDH1^+^
Melanoma	CD44^+^/CD15^+^/CD117^+^/CD34^+^/CD20^+^/ALDH1^h^
Pancreas	CD44^+^/CD133^+^/CD24^+^/EpCam^+^
Prostate	CD44^+^/CD133^+^/ 2 1^h^integrin∗∗∗

∗There are innumerable CSC phenotype combinations that depend on the histological type and the recurrence rate. ∗∗Also, designated as breast cancer resistance protein or ATP-binding cassette transporter G2. ∗∗∗Is the noncovalent heterodimer CD49b/CD29.

## Data Availability

This is a review article; therefore, there is no data availability.
